# miR-198 Inhibits HIV-1 Gene Expression and Replication in Monocytes and Its Mechanism of Action Appears To Involve Repression of Cyclin T1

**DOI:** 10.1371/journal.ppat.1000263

**Published:** 2009-01-16

**Authors:** Tzu-Ling Sung, Andrew P. Rice

**Affiliations:** Department of Molecular Virology and Microbiology, Baylor College of Medicine, Houston, Texas, United States of America; Northwestern University, United States of America

## Abstract

Cyclin T1 is a regulatory subunit of a general RNA polymerase II elongation factor known as P-TEFb. Cyclin T1 is also required for Tat transactivation of HIV-1 LTR-directed gene expression. Translation of Cyclin T1 mRNA has been shown to be repressed in human monocytes, and this repression is relieved when cells differentiate to macrophages. We identified miR-198 as a microRNA (miRNA) that is strongly down-regulated when monocytes are induced to differentiate. Ectopic expression of miR-198 in tissue culture cells reduced Cyclin T1 protein expression, and plasmid reporter assays verified miR-198 target sequences in the 3′ untranslated region (3′UTR) of Cyclin T1 mRNA. Cyclin T1 protein levels increased when an inhibitor of miR-198 was transfected into primary monocytes, and overexpression of miR-198 in primary monocytes repressed the normal up-regulation of Cyclin T1 during differentiation. Expression of an HIV-1 proviral plasmid and HIV-1 replication were repressed in a monocytic cell line upon overexpression of miR-198. Our data indicate that miR-198 functions to restrict HIV-1 replication in monocytes, and its mechanism of action appears to involve repression of Cyclin T1 expression.

## Author Summary

Monocytes do not support HIV-1 replication, in part because they do not express adequate levels of essential cellular cofactors that mediate steps in the viral replication cycle. Monocytes become permissive for viral replication upon differentiation to macrophages, indicating that cellular cofactors are induced during the differentiation process. One such cofactor is Cyclin T1, which is not expressed in monocytes and is expressed at high levels following macrophage differentiation. Cyclin T1 functions to greatly stimulate the amount of HIV-1 produced in the infected cell. We identified a microRNA (miRNA) named miR-198 that represses the expression of Cyclin T1 in monocytes. miRNAs block expression of proteins by binding to messenger RNAs and preventing their translation by ribosomes. The expression levels of miR-198 are greatly reduced in macrophages, and this appears to allow translation of Cyclin T1 mRNA and expression of Cyclin T1 protein. Our study indicates that this miRNA restricts HIV-1 replication in monocytes. We think that it is possible, if not likely, that additional miRNAs in monocytes also restrict HIV-1 replication by repressing other essential cellular cofactors.

## Introduction

Productive transcription of eukaryotic protein-coding genes requires a processive form of RNAP II to overcome pauses resulting from negative elongation factors. The positive transcription elongation factor, P-TEFb, plays a critical role in converting RNAP II to a processive enzyme through phosphorylation of the C-terminal domain of RNAP II and negative elongation factors [1],[2]. P-TEFb is composed of Cyclin-dependent kinase 9 (CDK9) as the catalytic subunit and Cyclin T1, T2, or K as the regulatory subunit [3],[4]. Although there are multiple Cyclin partners for CDK9, Cyclin T1-containing P-TEFb (Cyclin T1/P-TEFb) is the major cellular form in cell types examined thus far and it has been studied extensively because of its involvement in HIV-1 gene expression [5]. The HIV-1 Tat transactivator protein recruits Cyclin T1/P-TEFb to the TAR RNA structure of viral transcripts, resulting in a switch from an abortive transcription process to a highly processive one, and this greatly enhances viral gene expression and is essential for viral replication [6].

Unlike Cyclins involved in cell cycle progression, the expression level of Cyclin T1 is usually independent of cell cycle stages. However, Cyclin T1 has been shown to be regulated in human peripheral blood lymphocytes (PBLs), primary CD4^+^ T cells, and monocytes/macrophages. In PBLs and CD4^+^ T cells, activation from a resting state results in a strong up-regulation of Cyclin T1 protein expression through a mechanism that involves post-transcriptional regulation [7]–[9]. Cyclin T1 expression is low in freshly isolated monocytes and increases significantly when cells are induced to differentiate into macrophages [10]. The induction of Cyclin T1 in macrophages correlates with a permissive state for HIV-1 replication, as monocytes do not support HIV-1 replication [11]. Additionally, Cyclin T1 protein expression is shut-off at late times of macrophage differentiation by proteasome-mediated proteolysis, but it can be re-induced by macrophage activation or HIV-1 infection [10],[12].

The increase in Cyclin T1 protein expression in monocytes plays an important role in macrophage differentiation, as an shRNA depletion of Cyclin T1 in a monocytic cell line prevents the up-regulation of over 20% of mRNAs normally induced when these cells are stimulated to differentiate into macrophages [13]. We have previously observed that although Cyclin T1 protein expression is very low in freshly isolated primary monocytes, Cyclin T1 mRNA levels are high, suggesting that translation of this mRNA may be actively suppressed in monocytes [10],[14]. Given the approximate 5 kb length of the Cyclin T1 3′UTR [15], a miRNA(s) may be responsible for this repression. MicroRNAs are non-coding small RNAs of about 22 nucleotides in length that function in metazoans through base-pairing preferentially with the 3′UTR of target mRNAs, resulting in translational inhibition or in some cases mRNA degradation [16]–[18]. A number of studies have linked miRNAs to the regulation of specific gene functions, cell fate transition, malignant transformation, and especially hematopoietic lineage commitment [19]–[21].

In this study, we examined the miRNA expression profile during the early stage of primary human monocytes differentiation into macrophages. This analysis identified miR-198 as a negative regulator of Cyclin T1 protein expression through targeting sequences in the 3′UTR of Cyclin T1 mRNA. We found that inhibition of miR-198 function in primary monocytes resulted in increased Cyclin T1 protein levels, and overexpression of miR-198 in differentiating monocytes repressed the normal Cyclin T1 up-regulation. Inhibition of Cyclin T1 up-regulation by miR-198 in a monocytic cell line also resulted in decreased HIV-1 proviral gene expression and HIV-1 replication, indicating that miR-198 possesses an anti-HIV-1 function that appears to function through the repression of an essential cellular cofactor.

## Results

### Identification of miRNAs as candidates for repressors of Cyclin T1 protein expression in monocytes

Cyclin T1 protein expression is induced by a post-transcriptional mechanism when human primary monocytes are cultured *in vitro* under conditions that allow macrophage differentiation [10]. Given the ∼4.6 kb 3′UTR in the Cyclin T1 mRNA (see below), we wished to examine changes in miRNAs expression during monocyte to macrophage differentiation, as miRNAs that are down-regulated during differentiation are candidates for repressors of Cyclin T1 protein expression. We isolated total RNA from both monocytes and macrophages allowed to differentiate *in vitro* from two healthy blood donors. A microarray platform was used to examine expression levels of 321 validated human miRNAs in these RNA preparations (see Materials and Methods).

**Figure 1:**
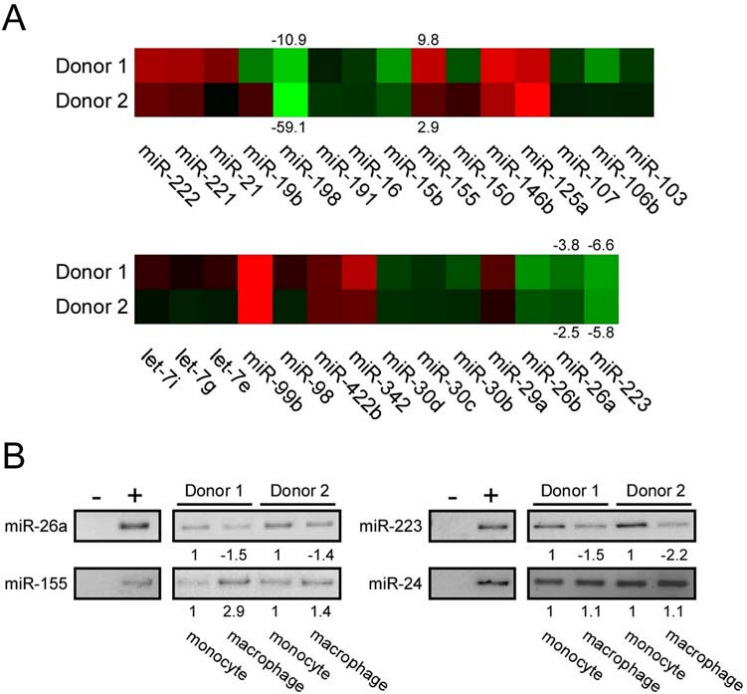
MiRNA expression profile during monocyte to macrophage differentiation. (A) Heat map for all differentially expressed miRNAs in two blood donors. Each color block represents the ratio of miRNA expression levels of monocytes to macrophages. Red indicates an induction and green indicates a reduction of expression in macrophages, with color intensity correlating with level of change. The fold-change of selected miRNAs is shown above or below the blocks. Positive number indicates an induction and negative number indicates a reduction. (B) Expression of miR-26a, miR-155, miR-223, and miR-24 were evaluated in semi-quantitative PCR assays. Equal amounts of RNA from the indicated RNA preparations from the two donors were used in end-point PCR assays. Negative controls (−) were reaction mixtures without RNA and positive controls (+) were reaction mixtures with human total heart RNA. Quantification of band intensities is shown below each PCR product.

Using criteria and statistical analysis described in Material and Method, we identified several miRNAs that were differentially expressed in monocyte and macrophages from both donors (Figure 1A). Of these miRNAs, nine were up-regulated (red in heat map) and 13 were down-regulated (green in heat map) in macrophages relative to monocytes. Thus, a total of 22 out of 321 miRNAs examined showed reproducible differential expression during monocyte to macrophage differentiation. To monitor the reliability of the miRNA microarray data, we selected four miRNAs for end-point RT-PCR analysis with commercially available primer sets. As shown in Figure 1B, increased amounts of PCR products for miR-155 were detected in RNA samples from macrophages relative to monocytes, in agreement with the microarray data which showed an induction of miR-155. Reductions in the levels of miR-26a and miR-223 in macrophages were detected in the PCR assays, again in agreement with the microarray data in which both of these miRNAs were down-regulated. Finally, the levels of miR-24 were similar between monocyte and macrophages in the PCR assays, in agreement with microarray data that showed a constant level of miR-24 expression. These results indicate that the microarray data are likely to be reliable in general.

### Identification of Cyclin T1 poly(A) signal

**Figure 2:**
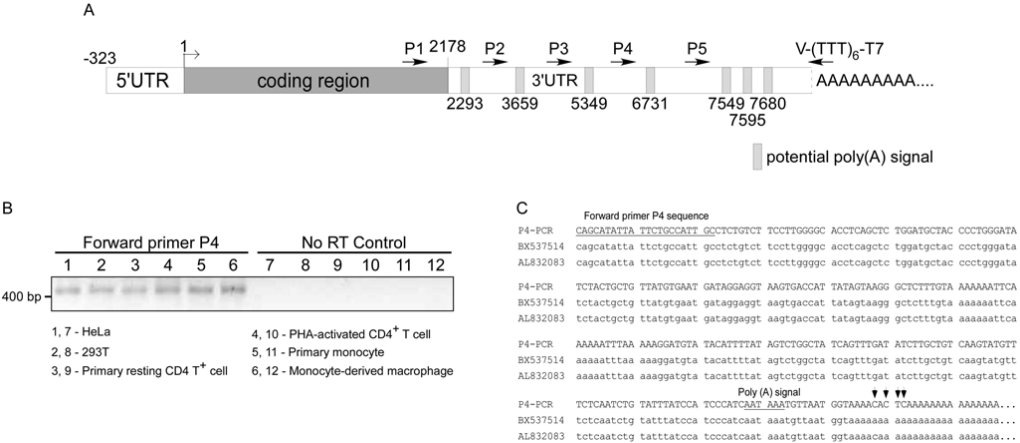
Cyclin T1 mRNA utilizes the fourth poly(A) signal downstream of the coding sequence. (A) A schematic representation of Cyclin T1 mRNA with 3′UTR end and potential poly(A) signals. The first nucleotide in the coding sequence is defined as +1. The indicated Reverse Transcription primer (T7-(TTT)_6_-V) contains T7 promoter sequence for PCR amplification and mixed nucleotides (V; A, C, and G) at the 3′ end for specific amplification from the beginning of the poly(A) sequences. Forward primers for PCR (P1 to P5) are indicated by forward arrows. (B) Reverse transcription was conducted with DNase I-treated RNA from HeLa cells, 293T cells, resting and activated primary CD4^+^ T cells, primary monocytes, and macrophages using primer T7-(TTT)_6_-V. PCR reactions with primers specific for T7 sequences and each utilized poly(A) signal (P1 to P5) is predicted to yield PCR products of ∼400 bp in length. An agarose gel as shown with products results for primer P4 and control reactions. (C) The sequence of the PCR product obtained with P4 primer shows sequence similarity with two cDNA clones, BX537514 (CoreNucleotide search) and AI832083 (EST search) in GenBank. Arrows indicate differences in nucleotide sequences between the product with the P4 primer and EST sequences.

To identify miRNAs that might repress Cyclin T1 mRNA translation, we wished to search Cyclin T1 mRNA sequences for potential target sites for the 13 miRNAs found to be down-regulated during monocyte differentiation. Cyclin T1 mRNA is ubiquitously expressed as a ∼8 kb transcript and because the coding sequence of Cyclin T1 only requires 2178 nucleotides and the 5′UTR is approximately 330 nucleotides in length, it has an extensive 3′ untranslated region (3′UTR) that has not been characterized [4],[22]. The alignment of human EST sequences and the full-length goat Cyclin T1 mRNA sequence suggest that the 3′UTR of human Cyclin T1 mRNA is contained within a single exon [15]. An analysis of the 8 kb sequence downstream of the Cyclin T1 stop codon revealed seven potential poly(A) signals (AATAAA) (Figure 2A). To determine the 3′UTR sequences for miRNA target predictions, we first characterized the poly(A) signal usage in the Cyclin T1 mRNA by RT-PCR.

RNA samples from cell lines, primary CD4^+^ lymphocytes, and monocytes/macrophages were examined to determine if alternative poly(A) signals are utilized for the Cyclin T1 3′UTR. Using an RT primer that anneals to the start of the poly(A) sequences and PCR primers specific for each poly(A) signal (P1 to P5, Figure 2A), we were able to amplify a discrete band of ∼400 bp only with primer P4 (Fig 2B). Extension times in PCR reactions were 45 seconds to maximize the expected ∼400 bp product that would be produced by the primer nearest to the major poly(A) site. These data suggest that the fourth poly(A) signal is utilized to produce the majority of Cyclin T1 mRNA in the cell types examined. The absence of PCR products in reactions without reverse transcription excludes the possibility of genomic DNA contamination in PCR reactions. A BLAST search revealed two cDNA clones with only three or four nucleotide differences with the P4-PCR product (Figure 2C), further supporting the conclusion that the fourth poly(A) signal is used predominantly. Based upon these results, we conclude that Cyclin T1 mRNA arises predominantly from utilization of the fourth poly(A) signal and its length is ∼7.1 kb (plus poly(A) sequences). Additionally, this analysis found no evidence of alternative poly(A) site selection in CD4^+^ lymphocytes and monocyte/macrophages. This deduced Cyclin T1 3′UTR was used for miRNA target site predictions.

### Ectopic expression of miR-198 down-regulates endogenous Cyclin T1 without affecting the mRNA levels

**Figure 3:**
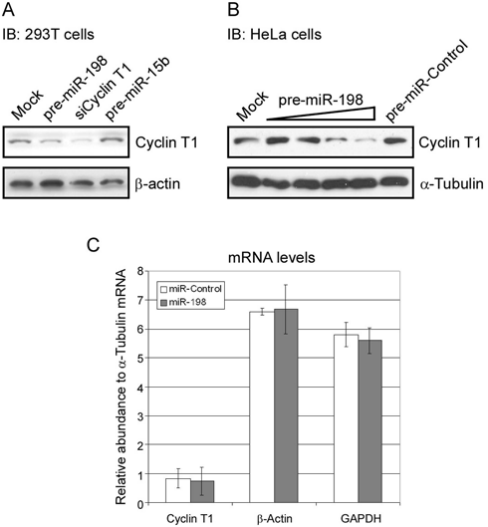
Ectopic expression of miR-198 down-regulates endogenous Cyclin T1 protein levels without affecting its mRNA levels. (A) 293T cells were transfected with 150 pmole of pre-miR-198, pre-miR-15b, or siRNA against Cyclin T1 (siCyclin T1) and examined for Cyclin T1 and β-actin protein expression at 72 hours after transfection. IB: immunoblot. (B) Increasing amounts of pre-miR-198 (6.25 pmole to 100 pmole) or pre-miR-Control (100 pmole) were transfected into HeLa cells. Cells were harvest at 72 hours post-transfection to examine the levels of Cyclin T1 and α-tubulin protein expression. (C) Cyclin T1 mRNA levels were examined by RT-real-time PCR and normalized to α-tubulin mRNA levels in pre-miR-198- and miR-Control-transfected HeLa cells. β-actin and GAPDH mRNA levels were also normalized to α-tubulin mRNA. The data shown here are means±SDs of three independent transfections.

We selected several down-regulated miRNAs, including miR-15b, miR-26a, and miR-198, for initial experiments. These three miRNAs were down-regulated >2-fold in our microarray data with monocytes/macrophages and each are predicted to have >five target sites in the Cyclin T1 mRNA 3′UTR with minimum free energies (mfe) less than -20 kcal/mol. In gain-of-function experiments, precursors for these miRNAs were transfecting into 293T cells and Cyclin T1 protein expression was examined in immunoblots. Cyclin T1 protein levels were reduced in 293T cells transfected with the miR-198 precursor (pre-miR-198) relative to mock-transfected cells, while miR-15b precursor (pre-miR-15b) had no significant effect (Figure 3A). Similar to the results with the pre-miR-15b transfection, the miR-26a precursor did not affect Cyclin T1 protein expression (data not shown). SiRNAs that target the Cyclin T1 coding region were used as a positive control for transfection, and these siRNAs successfully knocked down Cyclin T1 protein expression.

To further validate the effect of miR-198 on Cyclin T1 protein expression, increasing amounts of pre-miR-198 were transfected into HeLa cells, and a dose-dependent reduction in Cyclin T1 protein level was seen (Figure 3B). A control miRNA precursor, pre-miR-Control, did not affect Cyclin T1 expression. Additionally, miR-198 did not affect Cyclin T1 mRNA levels, which remained constant in cells transfected with pre-miR-198 (Figure 3C). The levels of β-actin and GAPDH mRNAs also remained unchanged following transfection of pre-miR-198, suggesting that miR-198 overexpression has no impact on overall mRNA levels. These data demonstrate that miR-198, a miRNA which is down-regulated during monocyte to macrophage differentiation, is capable of down-regulating Cyclin T1 protein expression. Because the ectopic expression of miR-198 reduces Cyclin T1 protein levels without affecting Cyclin T1 mRNA levels, it is likely that miR-198 acts through translational inhibition as is common for miRNAs.

### Identification of miR-198 target sites in the 3′UTR of Cyclin T1 mRNA

**Figure 4:**
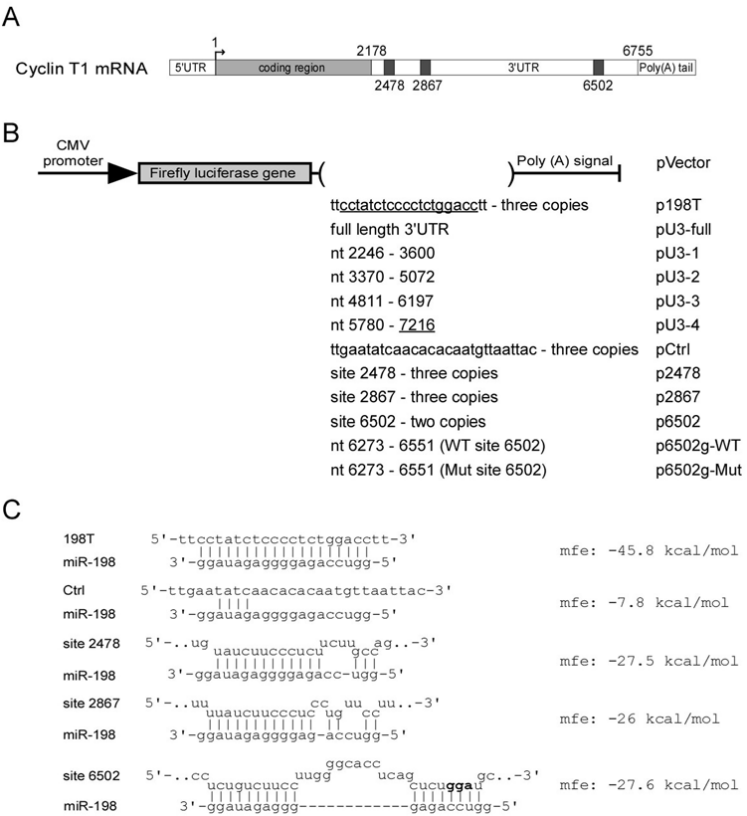
miR-198 target sequences in the Cyclin T1 mRNA 3′UTR. (A) Schematic representation of Cyclin T1 mRNA. Three predicted miR-198 target sequences, site 2478, site 2867, and site 6502, are shown and named according to the position of their first nucleotide in Cyclin T1 mRNA. (B) The indicated luciferase reporter plasmids based upon the CMV immediately-early promoter were constructed by inserting the indicated sequences between the firefly luciferase gene and poly(A) signal. pU3-4 contains additional sequences not included in Cyclin T1 3′UTR and is indicated with underlined number. (C) Predicted binding structures and energies (RNAhybrid) between miR-198 and the three predicted target sequences and control sequences as shown. The three nucleotides in site 6502 in bold font were mutated from gga to cct in p6502g-Mut.

**Figure 5:**
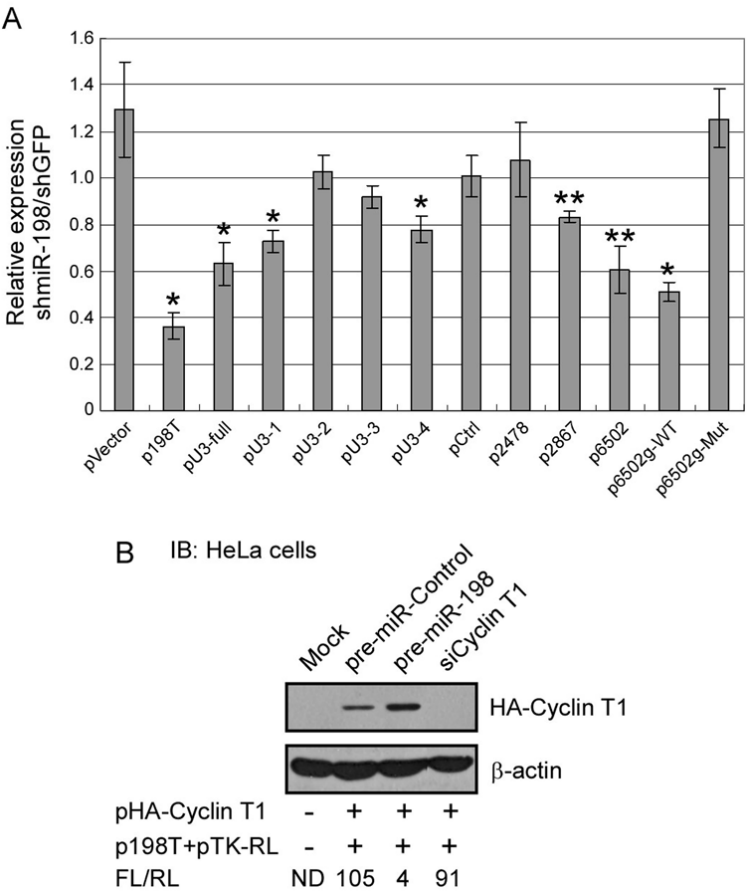
Identification of functional miR-198 target sites in Cyclin T1 3′UTR. (A) Reporter plasmids described in Figure 4B were co-transfected with a renilla luciferase internal control reporter (thymidine kinase promoter) into shGFP- or shmiR-198-expressing HeLa cell pools. Luciferase activities for firefly and renilla were measured 24 hours post-transfection. Relative luciferase activities (firefly luciferase activity by renilla luciferase activity, FL/RL) were measured for each plasmid. Relative expression was obtained after normalizing of FL/RL in shmiR-198-expressing cells to that in shGFP-expressing cells transfected. The data shown here are means±SDs of three independent transfections. * Students t-test P-value <0.001 compared to pVector. ** Students t-test P-value <0.001 compared to pCtrl. (B) An expression plasmid for HA-Cyclin T1, the p198T firefly luciferase reporter plasmid (Figure 4B) and a TK-renilla luciferase plasmid were co-transfected into HeLa cells with either pre-miR-control, pre-miR-198 or siRNAs against Cyclin T1. Cell extracts were prepared at 24 hours post-transfection and were used for luciferase assays and an immunoblot. The indicated ratios of firefly luciferase to renilla luciferase (FL/RL) was determined.

We found that miR-198 is predicted to have >10 potential target sites in the 3′UTR of Cyclin T1 mRNA with mfe lower than −20 kcal/mol (see Materials and Methods). To determine if the Cyclin T1 3′UTR can be regulated by miR-198, we inserted the full length 3′UTR between the luciferase coding sequence and the poly(A) signal of the pGL3 firefly luciferase vector driven by CMV immediate early promoter (pU3-full, Figure 4B). An shRNA vector was generated to express miR-198 (shmiR-198) from the pCL-based retroviral vector. An shRNA designed to target green fluorescence protein, shGFP, was also generated as a control. Pools of HeLa cells expressing shGFP or shmiR-198 were generated by infection of cells with shRNA retroviruses and subsequent puromyocin selection. Reporter plasmids were then transfected into shGFP- or shmiR-198-expressing HeLa cells along with a renilla luciferase reporter plasmid (pTK-RL) as an internal control for transfection efficiency. Additionally, p198T, which contains sequences with perfect complementarity to miR-198, was included as a positive control that should be repressed through an siRNA pathway. As shown in Figure 5A, the relative expression of the positive control p198T was repressed in cells expressing shmiR-198 relative to cells expressing shGFP, showing more than a 60% reduction. The parental pVector expressed at a slightly higher level in shmiR-198-expressing cells. Expression of pU3-full was also repressed by ∼40%, suggesting that there are miR-198 target sites in the Cyclin T1 3′UTR.

To identify the locations of these target sites, we divided the full-length 3′UTR into four overlapping fragments (U3-1 to U3-4) and inserted them into to the pGL3 vector (pU3-1 to pU3-4, Figure 4A and 4B). Only expression from the pU3-1 and pU3-4 reporters showed a statistically significant repression in shmiR-198-expressing cells, demonstrating a 30% and a 20% reduction for pU3-1 and pU3-4, respectively (Figure 5A). It appears therefore that target sites for miR-198 reside in these two fragments, consistent with recent findings that miRNA target sites are generally locate away from the center of a 3′UTR [23].

There were three predicted target sites in fragment U3-1 and U3-4 with mfe<−25 kcal/mol, and they were designated site 2478, site 2867, and site 6502 according to their locations in the Cyclin T1 mRNA (Figure 4A, first nucleotide in coding sequence defined as +1). All three predicted targets show extensive of complementarity with miR-198 at their 5′ ends. However, site 6502 contains additional eight nucleotides at its 3′ end complementary to the miR-198 seed sequence (Figure 4C), which closely resembles the structure of the 5′-dominant canonical miRNA target site [24]. Expression of reporter plasmids containing two or three copies of site 2478 (p2478), site 2867 (p2867), or site 6502 (p6502) in the 3′UTR of the luciferase gene was assayed in cells expressing either shmiR-198 or shGFP (Figure 5A). A control reporter plasmid, pCtrl, which contains three copies of unrelated sequences, expressed at similar levels in both shmiR-198- and shGFP-expressing cells. Expression of p2867 was reduced a relatively modest 20% in shmiR-198-expressing cells, while expression of p2478 was unaffected perhaps due to a sub-optimal seed sequence. Expression of p6502 was significantly repressed by shmiR-198, showing a 40–50% reduction in shmiR-198-expressing cells relative to control cells. Therefore, site 2867 in fragment U3-1 and especially site 6502 in fragment U3-4 appear to be responsible for repression mediated by miR-198 in Cyclin T1 mRNA 3′UTR. Although both of the sites have similar binding energies, site 6502 showed greater sensitivity to shmiR-198 when compared to site 2867. When site 6502 along with its adjacent sequences was clone into the reporter plasmid (p6502g-WT, Figure 4B) and assayed, a ∼50% reduction was observed in shmiR-198-expressing cells relative to shGFP-expressing cells (Figure 5A). In addition, mutation of three nucleotides in the site 6502 which are complementary to the mRNA seed sequence abolished the inhibition by shmiR-198 (Figures 4C and 5A), strongly suggesting the direct targeting of miR-198 to site 6502 and highlighting the importance of the seed match present in site 6502 during miRNA-mediated repression.

To verify that repression of Cyclin T1 protein expression by miR-198 requires target sequences in the Cyclin T1 3′UTR, we carried out a plasmid transfection experiment with a HA-tagged Cyclin T1 expression plasmid lacking the Cyclin T1 3′UTR. The HA-Cyclin T1 plasmid was co-transfected with a precursor for either miR-198 (pre-miR-198) or a pre-miR-Control. To verify that the transfected pre-miR-198 was biologically active, we co-transfected the p198T luciferase control plasmid (Figure 4C) that is repressed by miR-198 by a siRNA pathway. Additionally, a renilla luciferase reporter plasmid was co-transfected to monitor transfection efficiency and siRNAs against Cyclin T1 were included in a control co-transfection. The siRNAs against the HA-Cyclin T1 were very effectively in reducing protein expression but had little effect on expression of the p198T reporter plasmid (Figure 5B). Although the pre-miR-198 was able to reduce expression of the p198T reporter plasmid about 20-fold relative to the pre-miR control, it had no inhibitory effect on Cyclin T1 expression and even appeared to stimulate Cyclin T1 expression. These data indicate that the repression of Cyclin T1 protein expression by miR-198 requires target sequences in the Cyclin T1 mRNA 3′UTR.

### miR-198 regulates Cyclin T1 protein expression during in vitro monocyte to macrophage differentiation

**Figure 6:**
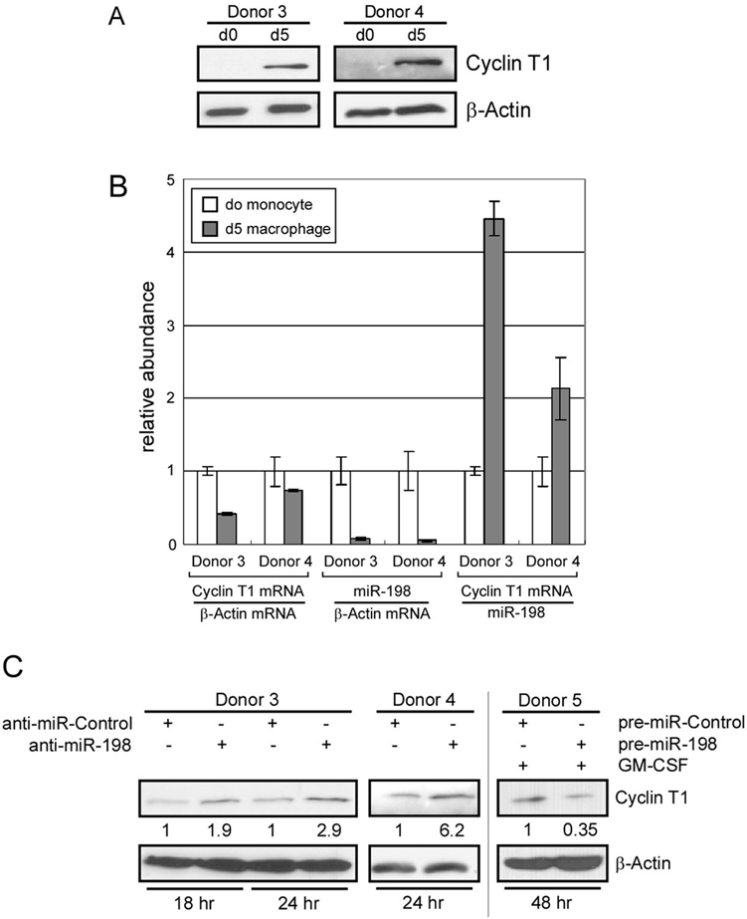
miR-198 functions to repress Cyclin T1 protein expression in primary monocytes. (A) Expression levels of Cyclin T1 and β-actin proteins in freshly isolated monocytes (d0) or macrophages allowed to differentiate for five days (d5) were examined in immunoblots for two donors. (B) Expression levels of Cyclin T1 mRNA relative to β-actin mRNA were measured by RT-real-time PCR assay; expression levels of miR-198 were measured using TaqMan MicroRNA assays. The relative abundance was calculated by normalizing of β-actin mRNA or miR-198 levels. Bars represent range in three measurements. (C) miR-198 inhibitor (anti-miR-198) or control inhibitor (anti-miR-Control) were transfected into freshly isolated monocytes from two donors (Donor 3, 4). miR-198 precursor (pre-miR-198) or control precursor (pre-miR-Control) were transfected into freshly isolated monocytes and differentiation was induced with GM-CSF treatment (Donor 5). Cells were harvested by direct lysis at times indicated after transfection and Cyclin T1 protein expression was examined in immunoblots. Quantification of Cyclin T1 protein levels is shown below each band (normalized to β-actin).

The data presented above demonstrate that miR-198 can target sequences in the Cyclin T1 mRNA 3′UTR and can repress expression of endogenous Cyclin T1 protein expression when ectopically expressed. To examine whether miR-198 regulates Cyclin T1 expression in primary monocytes, we isolated primary monocytes and macrophages from two donors (Donor3 and Donor4) and first re-examined the correlation between miR-198 expression and Cyclin T1 protein levels. In agreement with our previous results [10],[14], Cyclin T1 protein levels were strongly up-regulated in macrophages relative to monocytes (Figure 6A). Additionally, although Cyclin T1 protein expression is induced in macrophages, Cyclin T1 mRNA levels were reduced from 20 to 60% when compared to levels on monocytes (Figure 6B), similar to our previous results [10],[14]. In agreement with the microarray data shown in Figure 1A, miR-198 levels were reduced in macrophages relative to monocytes, showing a 14-fold reduction in Donor3 and a 19-fold reduction in Donor4. Although both Cyclin T1 mRNA and miR-198 levels were decreased in macrophages, the ratio between Cyclin T1 mRNA and miR-198 increased in macrophages. This observation indicates that in macrophages there are more copies of Cyclin T1 mRNA for each copy of miR-198.

In loss-of-function experiments, we examined the effects of a miR-198 inhibitor on Cyclin T1 expression in monocytes. We transfected a chemically modified single-stranded nucleic acid inhibitor of miR-198 (anti-miR-198) into freshly isolated monocytes. As shown in Figure 6C, anti-miR-198 was able to induce Cyclin T1 levels from two- to six-fold in two donors examined (Donor 3, 4). In an additional gain-of-function experiment, transfection of pre-miR-198 to overexpress miR-198 in monocytes induced to differentiate with GM-CSF treatment resulted in about a 3-fold decrease in Cyclin T1 protein levels (Figure 6C, right panel). The effects of miR-198 on Cyclin T1 expression are consistent with recent quantitative proteomic studies that have shown that in most cases miRNAs influence proteins levels about 1.5- to 2-fold [25],[26]. These data in primary monocytes further support the proposal that miR-198 plays a role in repressing Cyclin T1 protein expression in monocytes, and the reduction in miR-198 levels during macrophage differentiation contributes to induction of Cyclin T1 protein expression. The induction of Cyclin T1 has been shown to be important for the program of gene expression in macrophages [13].

### miR-198 represses HIV-1 proviral expression and HIV-1 replication

**Figure 7:**
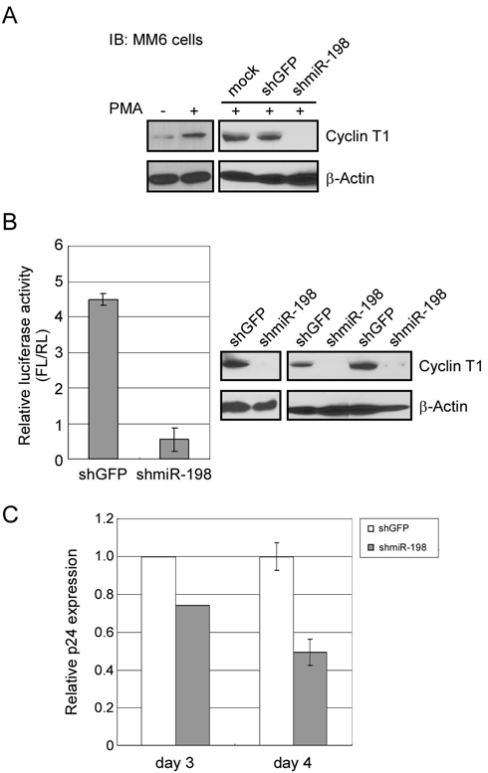
Down-regulation of Cyclin T1 by miR-198 inhibits HIV-1 proviral gene expression and HIV-1 replication. (A) Left panel: Immunoblot analysis of Cyclin T1 levels in MM6 cells treated with or without PMA for 48 hours. Right panel: MM6 cells were mock-transfected or transfected with shGFP- or shmiR-198-expressing plasmids and then treated with PMA. Cells were harvested 48 hours after PMA treatment and examined for Cyclin T1 expression. β-actin expression was analyzed as a loading control. (B) A HIV-1 NL4-3 firefly luciferase proviral reporter plasmid was co-transfected with shGFP- or shmiR-198-expression plasmids and a renilla luciferase reporter internal control plasmid into MM6 cells. Cells were treated with PMA immediately after transfection, and cell extracts were prepared 48 hours later and divided into two equal portions for dual-luciferase assays and immunoblot analyses. Relative luciferase activity was determined by normalizing firefly luciferase activity to renilla luciferase activity. Two independent transfection experiments were performed; in one experiment transfections were performed in duplicate. For immunoblots to examine Cyclin T1 expression, panel on left represents the single transfection experiment, while panel on right represents the duplicate transfection experiment. Luciferase assays shown are the average of the three transfections with bars representing the range in the three measurements. C. MM6 cells were transfected with shGFP- or shmiR-198-expression plasmids in two independent experiments. Cultures were treated with PMA immediately after transfection and one day later were infected with HIV-1 strain SF_162_. Expression of p24 in culture supernatants was measured at three days post-infection in experiment 1 and at four days post-infection in experiment 2. The data in experiment 2 show the means±SD of infections carried out in triplicate in the control (shGFP) or shmiR-198 plasmid.

In addition to cellular genes that are dependent on Cyclin T1 for expression, HIV-1 Tat-mediated gene expression is highly dependent upon Cyclin T1. We therefore examined if down-regulation of Cyclin T1 by miR-198 could result in decreased HIV-1 proviral expression. We used a promonocytic cell line, Mono Mac 6 (MM6) for these experiments, as we previously determined that this cell line is a useful model to investigate the role of Cyclin T1 function in monocyte differentiation [13]. Cyclin T1 is up-regulated in MM6 cells treated with PMA for 48 hours (Figure 7A, left panel), similar to its induction during primary monocyte to macrophage differentiation. Ectopic expression of miR-198 in MM6 cells from a transfected shmiR-198 plasmid, followed by PMA treatment, largely prevented Cyclin T1 up-regulation (Figure 7A, right panel). In contrast, transfection of a control shGFP plasmid resulted in similar Cyclin T1 levels as those seen in mock-transfected cells.

To examine if HIV-1 proviral gene expression is affected by the down-regulation of Cyclin T1 by miR-198, a HIV-1 NL4-3 luciferase proviral reporter plasmid was co-transfected with shGFP or shmiR-198 plasmid into MM6 cells. A renilla luciferase reporter plasmid, pTK-RL, was also co-transfected as an internal control. Cells were treated with PMA, harvested 48 hours after transfection, and divide into two equal portions for luciferase assays and immunoblot analysis. The results of two independent experiments are shown in Figure 7B; in one of these experiments, duplicate transfections were performed. Luciferase assays revealed that expression of the HIV-1 proviral reporter plasmid was approximately nine-fold lower in shmiR-198-expressing cells than in shGFP-expressing cells, and the results of immunoblots indicated that this reduction correlated with decreased Cyclin T1 protein levels. These data suggest that miR-198 is capable of repressing HIV-1 proviral expression by targeting its cellular cofactor Cyclin T1.

To examine if HIV-1 replication is also repressed by ectopic expression of miR-198, MM6 cells were transfected with a shGFP control or shmiR-198 plasmid and cells were treated with PMA immediately after transfection. Cultures were infected with M-tropic HIV-1 strain SF_162_ after one day of PMA treatment and p24 expression in culture supernatants was measured at either three or four days post-infection in two independent experiments (Figure 7C). A 25% reduction in p24 expression was observed when expression was examined at three days post-infection, while a 50% reduction was observed when p24 expression was examined at four days post-infection. The greater inhibition of proviral reporter plasmid expression by miR-198 in Figure 7B than in the HIV-1 infection in Figure 7C is likely the result of transfection efficiency. In the proviral reporter plasmid experiment, both the reporter plasmid and the miR-198 shRNA vector were co-transfected and it is therefore likely that the majority of transfected cells contained both plasmids. In the HIV-1 infection experiment, it is likely that a significant portion of infected cells were not transfected with the miR-198 shRNA vector. The data in Figure 7C further suggest that miR-198 is capable of repressing HIV-1 replication through down-regulation of the essential cofactor Cyclin T1.

### Effects of HIV-1 infection on miR-198 expression in macrophages

**Figure 8:**
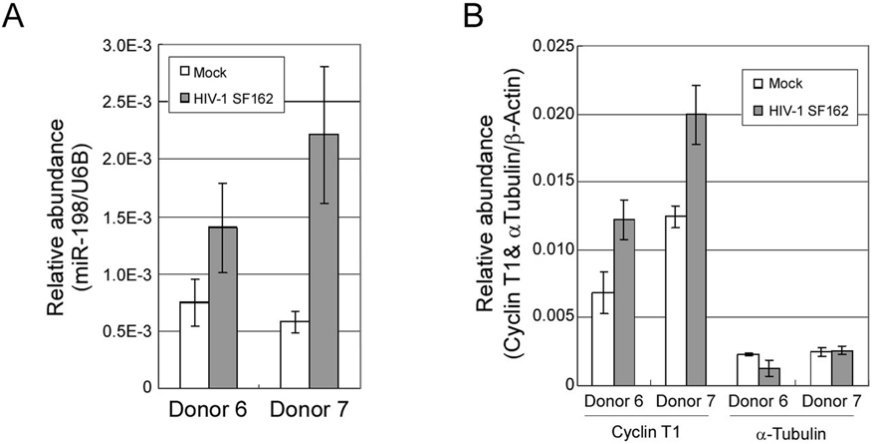
HIV-1 infection of primary macrophages affects miR-198 as well as Cyclin T1 mRNA levels. Macrophages were infected with HIV-1 SF162 strain for seven days and total RNA was isolated and assayed for: (A) expression levels of miR-198; (B) Cyclin T1 and α-tubulin mRNAs. U6B snRNA was used for normalization of miR-198 and β-actin mRNA was used for normalization of mRNAs. Bars represent the range in the three measurements.

It has been reported that miRNA expression shows distinct patterns in HIV-1 provirus plasmid-transfected HeLa cells [27]. Therefore, we wished to examine if HIV-1 infection affects miR-198 expression in macrophages. Macrophages isolated from healthy blood donors were allowed to differentiate for four days, and were infected with a M-tropic HIV-1 SF162 strain for seven days, and RNA was isolated for miR-198 quantification. Measurements of p24 expression were performed at day four and seven post-infection to verify productive infections (data not shown). As shown in Figure 8A, miR-198 levels increased modestly to 1.9-fold in Donor 6 and 3.8-fold in Donor 7 when normalized to U6B snRNA. These data suggest that HIV-1 infection up-regulates miR-198 levels in macrophages. Cyclin T1 mRNA levels also increased 1.8-fold in Donor 6 and 1.6-fold in Donor 7, while α-tubulin mRNA levels remained the same (Figure 8B). Cyclin T1 protein expression is induced in HIV-1-infected macrophages, and this has been shown to involve an inhibition of proteasome-mediated proteolysis of Cyclin T1 [12]. The data shown on Figure 7B indicates that a relatively modest increase in Cyclin T1 mRNAs may contribute to the induction in Cyclin T1 protein expression in infected macrophages. The significance in the modest increase in miR-198 levels in HIV-1 infected macrophages remains to be elucidated.

## Discussion

Our finding that miR-198 represses Cyclin T1 protein expression in monocytes provides mechanistic insight into previous observations that translation of Cyclin T1 mRNA is inhibited in these cells [10],[14]. Because Cyclin T1 is a component of P-TEFb, a general RNA polymerase II elongation factor, miR-198 is likely to broadly regulate gene expression in monocytes through its own targeting functions and through indirect effects on Cyclin T1-dependent genes. It is important to note that P-TEFb function is not deficient in monocytes, as these cells express high levels of Cyclin T2, an alternative Cyclin subunit of P-TEFb [13],[28]. Other miRNAs have been shown to target transcription factors and are also capable of broadly regulating gene expression through indirect mechanisms. For example, miRNA lin-4 targets lin-14 in *C. elegans* [29],[30] and in neuronal cells, miR-124 targets the RNAP II CTD phosphatase SCP1, a component of the REST transcription repressor complex [31].

### miR-198 and HIV-1 replication in monocytes

Freshly isolated monocytes do not support viral replication and must undergo a program of macrophage differentiation to become permissive for HIV-1 replication [11]. Viral entry is not limiting in monocytes, but reverse transcription and nuclear import of the pre-integration complex are defective [32],[33]. Because Tat is essential for HIV-1 replication [6] and Cyclin T1 is a critical Tat cofactor, miR-198 can function in monocytes as an additional repressor of viral gene expression and replication (Figure 6B and 6C). MiRNAs have previously been reported to regulated Tat function, as the miRNA cluster miR-17/92 is capable of inhibiting HIV-1 replication through repression of the histone acetyltransferase PCAF that also participates in Tat function [34].

We observed that HIV-1 infection of macrophages modestly induces both miR-198 and Cyclin T1 mRNA expression levels (Figure 7). Because HIV-1 infection induces Cyclin T1 protein expression in macrophages [12], it is possible that following up-regulation of Cyclin T1 mRNA and protein by infection, a cellular negative-feed back loop is activated that results in elevated levels of miR-198 and a subsequent dampening of the induction of Cyclin T1.

### Down-regulation of miR-198 and up-regulation of Cyclin T1 during monocyte differentiation

The up-regulation of Cyclin T1 protein expression is an important event during monocyte differentiation, as shRNA depletions and transcriptional profiling have shown that Cyclin T1 is required for expression of more than 20% of the mRNAs that are induced during the differentiation program [13]. Because miR-198 restricts Cyclin T1 expression in monocytes, this miRNA may contribute to the prevention of a transcriptional program of differentiation. The reduction of expression of miR-198 is therefore likely to be important for monocyte differentiation. Furthermore, genes involved in immune responses are overrepresented in the set of Cyclin T1-dependent mRNAs in macrophages [13], suggesting that proper macrophage function may require the down-regulation of miR-198. A topic for future research will be the study of signals and mechanisms that lead to the down-regulation of miR-198 in macrophages.

It is notable that Cyclin T1 mRNA levels are reduced ∼30–60% when Cyclin T1 protein levels are up-regulated following monocyte differentiation (Figure 5) [10],[14]. The explanation for this phenomenon is not clear, but it is possible that transcription of the Cyclin T1 gene is not increased during differentiation, as the promoter for Cyclin T1 appears to be constitutively active [22]. The active translation of Cyclin T1 mRNA following the relief of repression by miR-198 may reduce the mRNA half-life, and if a transcriptional induction of the Cyclin T1 gene does not occur, a decrease in the amount of Cyclin T1 mRNA will result.

### Expression pattern of miR-198 and other potential mRNA targets

miR-198 is in the 3′UTR (exon 11) of FSTL1, a gene that has recently been shown to enhance inflammatory cytokine production in a monocytic cell line after mitogen stimulation [35]. The correlation between the FSTL1 transcript and miR-198 levels has not yet been established, but FSTL1 transcripts are not detectable in peripheral blood leukocytes [36], suggesting that the processing of the FSTL1 primary transcript for protein production or miR-198 might be mutually exclusive.

Little information is available concerning potential functions and mRNA targets for miR-198. Although expression in monocytes was not examined in a previous study in 40 normal human tissues, miR-198 expression was found to be generally low and restricted to only a few tissues [37]. In hepatic tumors, expression of miR-198 was found to be down-regulated relative to normal liver parenchyma [38]. A genetic analysis identified a SNP in the miR-198 gene that has a nominally significant allelic association with schizophrenia [39]. We used both PicTar [40] and TargetScan [41],[42] to identity mRNAs that are predicted to be targets of miR-198. This analysis identified 41 mRNAs in common between the two programs that are potential targets for miR-198. However, no common features from the proteins encoded by these mRNAs were apparent. An intriguing predicted target for miR-198 is in the 3′UTR of PTEN, a negative regulator of the PI3-kinase pathway, and PTEN is involved in LPS signaling in monocytes/macrophages [43]. Interestingly, the HIV-1 Tat protein has been shown to decrease expression of PTEN in primary human macrophages [44], which is consistent with our findings that HIV-1 infection induces miR-198 expression in macrophages (Figure 8). Because overexpression of PTEN enhances HIV-1 expression [45], it is possible that the anti-HIV-1 function of miR-198 involves targeting cellular cofactors in addition to Cyclin T1.

We note that miR-223,which has been shown to target HIV-1 in the 3′ end of the viral genome and repress its expression [46], was identified in our miRNA profiling as a miRNA that is down-regulated during macrophage differentiation (Figure 1). This further emphasizes the power of miRNA profiling in identifying miRNAs associated with specific processes. Finally, given the ∼5 kb of 3′UTR sequence in Cyclin T1 mRNA, it is possible if not likely that miRNAs in addition to miR-198 are involved in repression of Cyclin T1 expression in monocytes and other cell types. Our miRNA profiling only assayed 321 miRNAs and given that it is estimated that there are >800 human miRNAs, it is possible that additional miRNAs that regulate Cyclin T1 in monocytes and other cell types await discovery.

## Materials and Methods

### Primary cell preparations

Peripheral blood mononuclear cells (PBMCs) were isolated from healthy blood donors (Gulf Coast Regional Blood Center, Houston, TX) by Isolymph density gradient centrifugation (Gallard-Schlesinger). Primary monocytes were isolated from PBMCs by negative selection using monocyte Isolation Kit II (Miltenyi Biotec). Macrophages from the same donors used for monocyte preparations were obtained by adherence of PBMCs to dishes in RPMI medium supplemented with 1% human serum for one hour as described previously [10]. Adhered cells were washed three times with phosphate buffered saline (PBS), incubated with complete RPMI medium (10% fetal bovine serum and 1% Penicillin-Streptomycin liquid, GIBCO) for two hours, washed three times with PBS, and cultured with complete RPMI medium containing GM-CSF (10 units/ml) for four to five days before RNA isolation. Purities of monocyte and macrophage preparations were evaluated by flow cytometry using CD14 as a monocyte marker and CD71 as a macrophage marker. The purity of monocyte and macrophage preparations was determined to be ≥ 92% (data not shown). Resting CD4^+^ T cells were isolated and activated as previous described [9].

### MiRNA microarray analysis, quantification, and target prediction

To prepare RNA samples for miRNA microarray or miRNA quantification, total RNA enriched with small RNAs was isolated using mirVana miRNA isolation kit (Ambion) according to the manufacturer's protocol. RNA preparations from Donor1 and Donor2 were sent to LC Sciences (Houston, TX) for miRNA microarray analysis using the μParaflo microfluidic chips and detailed process can be found at http://www.lcsciences.com. Briefly, probes for miRNAs were *in situ* synthesized on chips using photogenerated reagent chemistry with repeats for each probe to allow statistical analysis. Version 7.1 arrays were used to detect 321 unique mature miRNAs. RNA samples from the same donor for comparison were labeled with Cy3 or Cy5 for hybridization, and the labeling was reversed between the same cell populations from the two donors. Quality control was done by hybridizing a group of control oligos to evaluate array quality as well as by mixing a fixed amount of 20-mer RNA oligos to samples as external controls. Data were processed by subtracting background and normalized the signals using a LOWESS filter [47]. A detectable transcript was defined by fulfilling the following criteria: (1) signal intensity >3×background standard deviation; (2) spot CV (standard deviation/signal intensity) <0.5; (3) signals from at least 50% of the repeating probes are above detection level. The ratio of the two colors of detected signals and p-values of the t-test were calculated. A cut-off at p-value < 0.01 was used to define a differentially expressed miRNA. A heat maps for all differentially expressed miRNAs was generated by Cluster [48]. The primary data can be accessed at http://www.bcm.edu/molvir/labs/herrmann-rice-lab/Rice-Herrmann_Pages-Data.htm.

End-point PCR analysis for miR-26a, miR-155, miR-223, and miR-24 were used to verify microarray results of Donor1 and Donor2 by mirVana qRT-PCR detection kit and primer sets (Ambion). A 25-cycle of PCR was preformed and the PCR products were resolved by gel electrophoresis using a 3.5% agarose gel to examine the ∼90 bp products. Quantification of band intensity was done by analysis of the gel pictures with ImageQuant (Molecular Dynamics). TaqMan MicroRNA assays (Applied Biosystems) were use to quantify mature miR-198, U6B snRNA, and U3B snRNA. Target prediction was done using primarily RNAhybrid [49] and also microInspector [50].

### Poly(A) signal usage of Cyclin T1 mRNA

Total RNA was isolated from cells using RNeasy mini kit (Qiagen) and treated with DNase I (Invitrogen) for reverse transcription using ThermoScript RT-PCR system (Invitrogen) and primer T7-(TTT)_6_-V (5′-TTGTAATACGACTCACTATAGGGC-TTTTTTTTTTTTTTTTTT-A/G/C-3′). PCR was performed using reverse primer for T7 and forward primers specific for each poly(A) signal (P1: 5′-gggccaatggtcacaacacg-3′, P2: 5′-TGTATATCCGAAGGGAAACAGC-3′, P3: 5′-GCTGAACTGTAGTGAAGCATCG-3′, P4: 5′-CAGCATATTATTCTGCCATTGC-3′, and P5: 5′-ATTGTTACATGAATACCTGG-3′). PCR products were resolved on a 2% agarose gel and bands of ∼400bp were extracted from the gel, purified with QIAquick gel extraction kit (Qiagen) for TOPO TA cloning (Invitrogen) for DNA sequencing.

### Ectopic expression of miR-198 and reporter assay

An shRNA strategy was utilized to express miR-198 (shmiR-198) or a control shRNA designed to target GFP (shGFP) from a pCL-based retroviral vector [51]. BOSC cells were used to generate viruses by co-transfecting shGFP or shmiR-198 plasmid and an amphotropic packaging vector using lipofectamine 2000 (Invitrogen). Viruses were harvested 24 hours after transfection for infection of HeLa cells for another 24 hours. Infected HeLa cells were then selected by puromyocin treatment (1 µg/ml) for two days to obtain cell pools expressing shGFP or shmiR-198. As quantified by TaqMan MicroRNA assays (Applied Biosystems), miR-198 was expressed >400-fold in cell pools expressing shmiR-198 relative to pools expressing shGFP.

Full-length or fragments of Cyclin T1 3′UTR, or copies of three predicted target sequences were inserted between the luciferase gene and the poly(A) signal of a pGL3-based firefly luciferase reporter driven by CMV immediate early promoter (Promega). The full-length Cyclin T1 3′UTR was cloned by PCR reactions using BAC clones as templates and extra ∼500bp genomic sequence downstream of the poly(A) signal was included for cloning into the luciferase reporter (pU3-full). Fragments of Cyclin T1 3′UTR were cloned by PCR reactions using pU3-full as template and primers containing *XbaI* site at the 3′ ends for subsequent cloning. Individual predicted target sequence was obtained by annealing complementary oligos containing copies of the predicted sequence and 3′ overhang for *XbaI* site ligation. Mutagenesis was done using QuikChange Site-Directed Mutagenesis Kit (Stratagene) according to manufacturer's instructions.

For reporter plasmid experiments, 25 ng of each firefly luciferase reporter plasmid was co-transfected into shGFP- or shmiR-198-expressing HeLa cell pools along with 10 ng of thymidine kinase promoter-driven renilla reporter plasmid (pTK-RL, control for transfection efficiency) using Lipofectamine 2000 (Invitrogen). To further boost miRNA expression, 1ug of shGFP- or shmiR-198 plasmids were also co-transfected. Cells were harvested 24 hours after co-transfection and luciferase activities were measured using Dual-Luciferase Reporter Assay System (Promega). Relative luciferase activity was calculated by dividing firefly luciferase activity by renilla luciferase activity (FL/RL). Relative expression was obtained by normalization to FL/RL in shGFP-expressing cells transfected with the same firefly reporter plasmid.

For the transfection experiment to examine the requirement of the Cyclin T1 3′UTR for repression by miR-198, pHA-Cyclin T1 [4] (50 ng), p198T (50 ng) and pTK-renilla luciferase (10 ng) were co-transfected into HeLa cells with pre-miR-Control (50 nmol), pre-miR-198 (50 nmol) or siCyclin T1 (50 nmol) in a 24-well culture dish using Lipofectamine (Invitrogen). Cells were harvested at 24 hours post-transfection and split into two portions for immunoblot analysis and dual-luciferase assays.

### MiRNA precursor/inhibitor transfections, Cyclin T1 immunoblots, and RT-real-time-PCR for cellular mRNAs

miRNA precursors (6.25 pmole to 100 pmole, Applied Biosystems) or an siRNA targeting Cyclin T1 coding region (Dharmacon) were transfected into HeLa or 293T cells (∼30% confluent at times of transfection) using oligofectamine (Invitrogen). Cells were harvested by direct lysis with 1×loading buffer for immunoblot analysis at 72 hours post-transfection. Independent transfections were performed in HeLa cells for total RNA isolation using RNeasy mini kit (Qiagen) and RNA were subjected to RT-real-time-PCR assays for quantification of Cyclin T1, β-actin, GAPDH, and α-tubulin mRNA levels using the Bio-Rad MyIQ single color detection system as previously described (Sung and Rice, 2006). Primer sequences designed by the Beacon Designer 2.0 (Premier Biosoft) are: β-actin-F 5′-AGCAAGCAGGAGTATGACGAGTC-3′, β-actin-R 5′-AGAAAGGGTGTAACGCAACTAAGTC-3′, GAPDH-F 5′-CGCCAGCCGAGCCACATC-3′, GAPDH-R 5′-AAATCCGTTGACTCCGACCTTCAC-3′. miR-198 inhibitor (anti-miR-198) or control miRNA inhibitor (anti-miR-Control) was transfected into primary monocytes (250 pmole inhibitors/3×10^6^ cells) obtained from healthy donors using the Amaxa Nucleofector system. Cells were harvested by direct lysis at 18 or 24 hours after transfection for immunoblot analysis to detect Cyclin T1 and β-actin protein expression. Pre-miR-198 or pre-miR-Control was also transfected with the Amaxa system into primary monocytes (250 pmole inhibitors/3×10^6^ cells) and cells were treated with GM-CSF to induce differentiation for 48 hours. Cyclin T1 protein levels were quantified by scanning X-ray films with Personal Densitometer SI and analyzed with ImageQuant (Molecular Dynamics), followed by normalization to β-actin protein levels. Antibodies for immunoblot were purchased from Santa Cruz Biotechnology.

### Transfections and HIV-1 infection in MM6 cells

The promonocytic cell line MM6 was cultured and transfected according to the protocols described at http://www.monocytes.de. Briefly, MM6 cells were propagated in RPMI 1640 (Invitrogen) supplemented with L-glutamine (2 mM, Invitrogen), penicillin-streptomycin (400 U/ml and 400 µg/ml, Invitrogen), 1×non-essential amino acids (Invitrogen), OPI media supplement (Sigma), and 10% certified fetal bovine serum tested for endotoxin (Invitrogen). Low passage MM6 cells were used and cells were kept at a concentration of no more than 5×10^6^/ml to maintain a low endogenous Cyclin T1 protein expression level. For HIV-1 wild type LTR-luciferase reporter assays, 5×10^6^ MM6 were washed with RPMI 1640 and incubated with 3 µg shGFP- or shmiR-198-expressing plasmids, 1 µg HIV-1 luciferase reporter plasmid, 1 µg pTK-RL, and 250 µg/ml DEAE dextran (Sigma) for 90 minutes. The HIV-1 luciferase reporter plasmid contains a deletion in the *env* gene, a replacement of the *nef* gene with firefly luciferase gene, and a deletion in the *vpr* gene. Cells and plasmids were mixed every 30 minutes by gently rocking the tube during incubation and then treated with 10% DMSO for 3 minutes. After washing out DMSO, cells were treated with PMA (10 µg/ml final concentration), harvested 48 hours later, and divided into two equal portions for dual-luciferase assays and immunoblot analyses as described above. HIV-1 infections with strain SF_162_ (100 TCID_50_ units per culture in 6 well dishes) were performed one day after PMA treatment. Measurements of p24 expression (RETROtek, ZeptoMetrix Corporation) in culture supernatants were performed at day three or four post-infection as indicated.

### HIV-1 infection of primary macrophages

Macrophages were allowed to differentiate for five days from monocytes and incubated with HIV-1 SF_162_ strain at 6000 TCID_50_ per 10 cm^2^ dish for two hours (37°C, 5% CO_2_) with intermittent shaking. Following virus adsorption, cells were washed three times with PBS. Cells were then supplied with complete RPMI medium and incubated for seven days to allow at least two rounds of infection. Measurements of p24 expression (RETROtek, ZeptoMetrix Corporation) at day four and day seven of infection were performed to verify productive infections. At day seven post-infection, cells were washed with PBS three times for total RNA isolation for miR-198 quantification and mRNA quantification as described above.
